# Identification of Neck Lymph Node Metastasis-Specific microRNA—Implication for Use in Monitoring or Prediction of Neck Lymph Node Metastasis

**DOI:** 10.3390/cancers15153769

**Published:** 2023-07-25

**Authors:** Yutaro Higashi, Kodai Nakamura, Ryota Takaoka, Mika Tani, Yusaku Noma, Kazuki Mori, Kota Yamashiro, Seiya Yokoyama, Tomofumi Hamada, Tsuyoshi Sugiura

**Affiliations:** 1 Department of Maxillofacial Diagnostic and Surgical Science, Field of Oral and Maxillofacial Rehabilitation, Graduate School of Medical and Dental Science, Kagoshima University, Kagoshima 890-8544, Japan; yutaro.higashi.b6@tohoku.ac.jp (Y.H.); pero-pulse-4.14@true.ocn.ne.jp (K.N.); k7506163@kadai.jp (R.T.); k1946287@kadai.jp (M.T.); k7794925@kadai.jp (Y.N.); k5538059@kadai.jp (K.M.); yama19@dent.kagoshima-u.ac.jp (K.Y.); 2 Division of Oral and Maxillofacial Oncology and Surgical Sciences, Tohoku University Graduate School of Dentistry, Sendai 980-8575, Japan; 3 Department of Pathology, Kagoshima University Graduate School of Medical and Dental Sciences, Kagoshima University, Kagoshima 890-8544, Japan; yokoyama@m3.kufm.kagoshima-u.ac.jp; 4 Department of Oral & Maxillofacial Surgery, Hakuaikai Medical Cooperation Sagara Hospital, Kagoshima 892-0833, Japan

**Keywords:** oral cancer, oral squamous cell carcinoma, microRNA, biomarker, tumor marker, metastasis, neck lymph node metastasis

## Abstract

**Simple Summary:**

There are no established biomarkers for oral squamous cell carcinoma (OSCC), and detection of neck lymph node metastases relies on imaging studies. Diagnosis and prediction using biomarkers, which are less invasive and simpler than imaging tests, could improve prognosis if lymph node metastases could be treated earlier. In this study, serum microRNAs were successfully used to diagnose primary neck lymph node metastases and predict late neck lymph node metastases. They reflected the presence of lymph node metastasis more accurately than serum squamous cell carcinoma antigens or pathological factors.

**Abstract:**

MicroRNAs (miRNAs) have attracted attention as non-invasive cancer biomarkers in various cancers; however, they have not been adequately investigated in oral squamous cell carcinoma (OSCC). This study investigated the diagnostic performance of serum-derived miRNAs at initial diagnosis for primary neck lymph node metastasis and the predictive performance for late neck lymph node metastasis based on long-term (up to approximately 8 years) follow-up of patients with OSCC. The expression of miRNAs in 40 patients with OSCC was quantified using real-time PCR (qPCR), and a comprehensive statistical analysis of the correlation of miRNA expression for primary and late neck lymph node metastases was performed. For the diagnosis of primary neck lymph node metastases, miR-423 and miR-125 were accurate. The miRNA index for primary metastasis diagnosis (miR-PM) calculated by regression analysis showed high diagnostic accuracy. The miR-5100 was useful for predicting late neck lymph node metastases. The miRNA index for late metastasis prediction (miR-LM) calculated using regression analysis showed high prediction accuracy. MiRNAs were useful for diagnosing primary neck lymph node metastases in OSCC and predicting late neck lymph node metastases. It may help to consider individualized treatment, including follow-up, surgical methods, and postoperative management.

## 1. Introduction

Oral and oropharyngeal cancer is the seventh most frequent type of cancer and the ninth most common cause of death worldwide. Approximately 710,000 cases and 359,000 deaths occur annually, with the Asian region in particular having a higher incidence and mortality rate of oral and oropharyngeal cancer than other regions [1]. Approximately 90% of oral and oropharyngeal cancers were diagnosed as squamous cell carcinomas (OSCC) by histology [2].

Lymph node (LN) metastasis is a major prognostic factor in cancer, especially neck LN metastasis, which occurs at the most common site of OSCC metastasis. Neck LN metastasis is an important prognostic factor with a 50% reduction in survival, and effective treatment is essential for disease control [3,4]. In the case of neck LN metastases, detecting metastases at the appropriate time is necessary, and surgery is suggested. In particular, late metastases, which occur in the neck LNs after surgical removal of the primary tumor, have a significant prognostic impact. The main reason for this phenomenon is the absence of effective tumor markers for OSCC; therefore, detection of late metastases is currently dependent on periodic imaging. The timing of diagnostic imaging often leads to delayed detection of metastases, which makes treatment more difficult and has a negative impact on prognosis. Even a relatively early stage of oral cancer (Stage I or II) without metastases is associated with a high risk of potential neck LN metastases. The National Comprehensive Cancer Network (NCCN) guidelines advocate prophylactic neck dissection according to the depth of invasion (DOI) of the primary tumor [5]. However, the positive rate of LN metastases with prophylactic dissection is approximately 30% [6], often resulting in surgery. Therefore, the indications for prophylactic neck dissection are still inconclusive.

Studies on the correlation between pathological grading of OSCC and neck LN metastasis to predict metastasis in the neck LN have been established. For instance, Jakobsson’s classification is a typical histopathological grading method [7]. Yamamoto et al. further classified modes of invasion by focusing on the spore morphology at the tumor host border and published the YK classification [8]. Yamamoto et al. reported that the YK classification correlates with neck LN metastasis, with YK-4C and YK-4D cases in particular having a higher frequency of neck LN metastasis and poorer prognosis [9]. Close et al. reported that many cases with neck LN metastasis had histopathological venous invasions [10]. Mascitti et al. reported that lymphatic invasion is an independent negative factor for neck LN metastasis [11]. Tumor budding represents a loss of cell cohesion and activation of infiltration and has been reported to be associated with LN metastasis and life expectancy [12]. There are also reports that epithelial–mesenchymal transition (EMT) of the primary tumor and VEGF expression patterns correlate with LN metastasis [13]. Histopathological findings are important prognostic factors; however, it is difficult to use them as objective indicators [14]. Therefore, the development of new and more accurate biomarkers to complement conventional prognostic factors is urgently needed.

miRNAs are 18–25 nucleotide single-stranded RNAs that regulate gene expression but do not encode proteins. They are associated with important functions in a variety of biological activities, including cell proliferation, differentiation, apoptosis, development, immune regulation, and aging [3]. miRNAs are protected from ribonuclease (RNase), making them stable and detectable in tissues and body fluids such as plasma and serum [4]. In different types of cancer, miRNA expression profiles are specific and changes in miRNA profiles are associated with characteristics of malignant transformation of cells [15]. In OSCC tissues and cell lines, several types of miRNAs have been reported to be altered and associated with metastasis [16]. We have previously reported that serum miRNAs can be used to diagnose OSCC [17].

In this study, based on long-term follow-up of patients with OSCC (up to approximately 8 years), we used previously reported OSCC-specific miRNA candidates to identify serum miRNAs associated with neck LN metastasis. The usefulness of miRNAs as biomarkers was also investigated using the diagnosis of primary neck LN metastasis and prediction of late neck LN metastasis as endpoints.

## 2. Materials and Methods

### 2.1. Study Design and Patients

In this study, 40 patients diagnosed with OSCC at the Department of Oral Surgery, Kagoshima University Hospital (Kagoshima, Japan) between 2016 and 2018 were recruited. Clinical data on each case, including age, sex, site, tumor size, and nodal status, were obtained from patient files. Staging was performed on all patients according to the criteria of the Tumor-Node-Metastasis (TNM) classification of malignant tumors by the International Union Against Cancer (The TNM Classification of Malignant Tumors, 8th Edition). The protocol for this research project was approved (code: 28-159, 5 September 2016; code: 220145, 16 January 2023) by the Ethics Committee of Kagoshima University. Written informed consent was obtained from all patients before the commencement of the study.

In this study, cases with neck LN metastasis at primary surgery were defined as primary neck LN metastasis. In addition to original late neck LN metastasis, cases in which neck dissection was performed at the time of primary surgery but metastasis were found in the contralateral neck LNs were also included and defined as late neck LN metastasis in a broad sense.

### 2.2. Blood Sample Collection

All blood samples from patients with OSCC were collected prior to treatment (14 days before surgery). Whole blood samples (10 mL) were collected in serum separator tubes (TERUMO, Japan) from all participants. For complete clotting, the tubes were allowed to stand at room temperature for 30 min, which was followed by centrifugation for 10 min at 1900× *g* at 4 °C. The upper serum fraction was recovered, and additional centrifugation for 10 min at 1600× *g* at 4 °C was performed to eliminate cell debris. Supernatants used as serum samples were stored at −80 °C until analysis. The level of serum squamous cell carcinoma (SCC) antigen was measured with chemiluminescence immunoassay using serum collected at the same time as the miRNAs. A cut-off value of 1.5 ng/mL was used, as defined by the National Cancer Center in Japan.

### 2.3. RNA Isolation and Quantitative Real-Time PCR

Using the miRNeasy Serum/Plasma Kit (Qiagen, Chatsworth, CA, USA), small RNA was isolated from 200 µL serum samples following the manufacturer’s instructions. The serum-derived miRNAs were subjected to real-time PCR. We selected 14 miRNAs on the basis of primer availability and literature review (Table S1).The expression levels of 14 selected miRNAs in each serum sample from 40 patients with OSCC were measured using a quantitative real-time RT-PCR method according to the manufacturer’s instructions using TaqMan^®®^ MicroRNA Assays (Applied Biosystems, Foster City, CA, USA) and TaqMan^®®^ Universal PCR Master Mix II (Applied Biosystems). PCR cycling conditions were as follows: 10 min at 95 °C for 1 cycle, followed by 45 cycles at 95 °C for 15 s, and 60 °C for 60 s. We used miR-16 as an internal control. As miR-16 is stable in blood samples, it has been used as a control miRNA in previously published studies [18,19,20]. The relative expression of target miRNA in each sample was calculated using the 2-ddCt method [21].

### 2.4. Pathological Sample Collection

Histopathological findings were based on the pathology report at the time of surgery. Pathology reports were reviewed and reported by at least two pathologists from the Department of Molecular Oral Pathology and Oncology, Kagoshima University Hospital, Japan. Decisions were made according to General Rules for Clinical and Pathological Studies on Oral Cancer, 2nd edition. Lymphatic and venous invasion were considered positive if tumor cells were found in the vessels. Perineural invasion is positive if tumor cells are in direct contact with nerve fibers. Lymph node metastasis was defined as the presence of tumor cells similar to primary tumor cells in the neck lymph nodes. The YK classification was used to classify the type of invasion [8]. This classification focuses on the morphology of the foci at the tumor-host interface between the endpoints of the Jakobsson and Willen classifications. The following classification was used: YK-1, defined as having a clear border; YK-2, defined as having a slightly weeping border; YK-3, defined as having an indistinct border and infiltrating small and large cancer foci; YK-4C, defined as having an indistinct border and infiltrating small cancer foci in a cordate pattern; and YK-4D, defined as having an indistinct border and diffuse infiltration without cancer foci. The typical histology that was determined to be lymphatic and vascular invasion and the representative histology for each of the YK classifications in the present study are provided in Supplementary Materials (Figure S1).

### 2.5. Statistical Analysis

The prognosis performance of candidate miRNAs was determined by calculating the area under the receiver operating characteristics (ROC) curve (AUC). Associations between miRNAs and primary and late neck LN metastasis were evaluated using cut-off values determined from the AUCs and Fisher’s exact tests. Relative expression levels of miRNAs between groups were compared using the Wilcoxon signed-rank test. A prediction model for miRNA index for primary metastasis diagnosis (miR-PM) and miRNA late metastasis prediction index (miR-LM) was constructed using linear regression. The Kaplan–Meier (KM) method was employed to analyze disease-free survival (DFS), and the log-rank method was used to compare differences between groups. These analyses were performed using JMP (SAS Institute Inc., Cary, NC, USA). Statistical significance was set at *p* < 0.05. The sample size calculation was performed using G*Power version 3.1.9.7. (Universität Kiel, Kiel, Germany).

## 3. Results

### 3.1. Clinicopathological Characteristics of Patients with OSCC

The characteristics of study participants and clinicopathological characteristics of patients with OSCC are presented (Table 1). The mean age of the patients was 67.3 years; 22 were male and 18 were female. The primary site of metastasis was the tongue in 21 patients, gingiva in 14, floor of the mouth in 4, and buccal mucosa in 1. Venous, lymphatic, and perineural invasions were present in 14, 3, and 7 patients, respectively. The YK classification was used for the type of invasion: YK-2 in 1 patient, YK-3 in 29 patients, YK-4c in 6 patients, and YK-4d in 4 patients. Twelve patients had primary neck LN metastases and 10 patients had late neck LN metastases.

### 3.2. Diagnosis of Primary Neck LN Metastases Using Single miRNA

**Figure 1:**
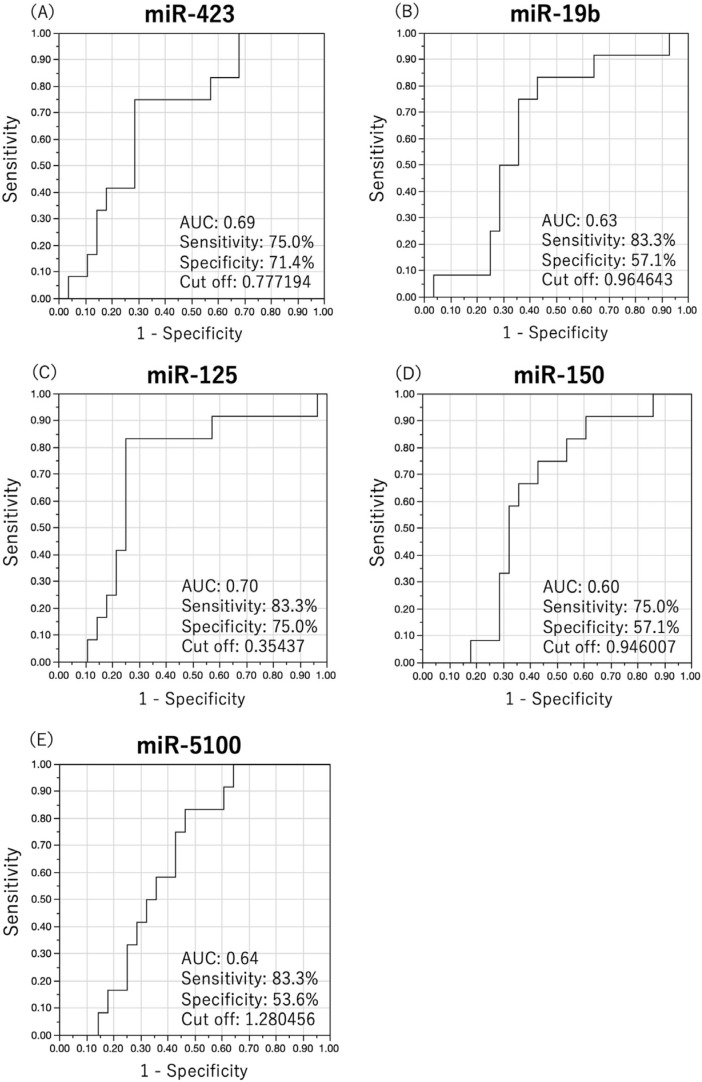
ROC analysis for primary neck lymph node metastases using a single miRNA. Diagnostic performance of each miRNA was investigated by determining the cut-off value using ROC analysis, with the detection of primary neck lymph node metastasis as the outcome. The ROC curves of five miRNAs with useful diagnostic performance are shown. miR-423 (**A**), miR-19b (**B**), miR-125 (**C**), miR-150 (**D**), and miR-5100 (**E**) miRNAs showed good diagnostic performance with an AUC of 0.6 or higher.

We have previously identified OSCC-specific miRNA candidates by comprehensive analysis of serum miRNAs [17]. From these OSCC-specific miRNAs, we attempted to identify neck LN metastasis-specific miRNAs. Using preoperative serum from patients with OSCC, we performed quantitative measurement of OSCC-specific miRNAs using real-time PCR. Cut-off values were determined using ROC analysis with detection of primary neck LN metastasis as the outcome. The diagnostic performance of each miRNA was examined based on the cut-off values from the analysis. The ROC curves of the five miRNAs with useful diagnostic performance are shown in Figure 1 and the results of each analysis are shown in Table 2. miR-423, miR-19b, miR-125, miR-150, and miR-5100 showed good diagnostic performance with AUC values of 0.6 or higher. Results for the other nine miRNAs are provided in Supplementary Materials (Figure S2).

**Figure 2:**
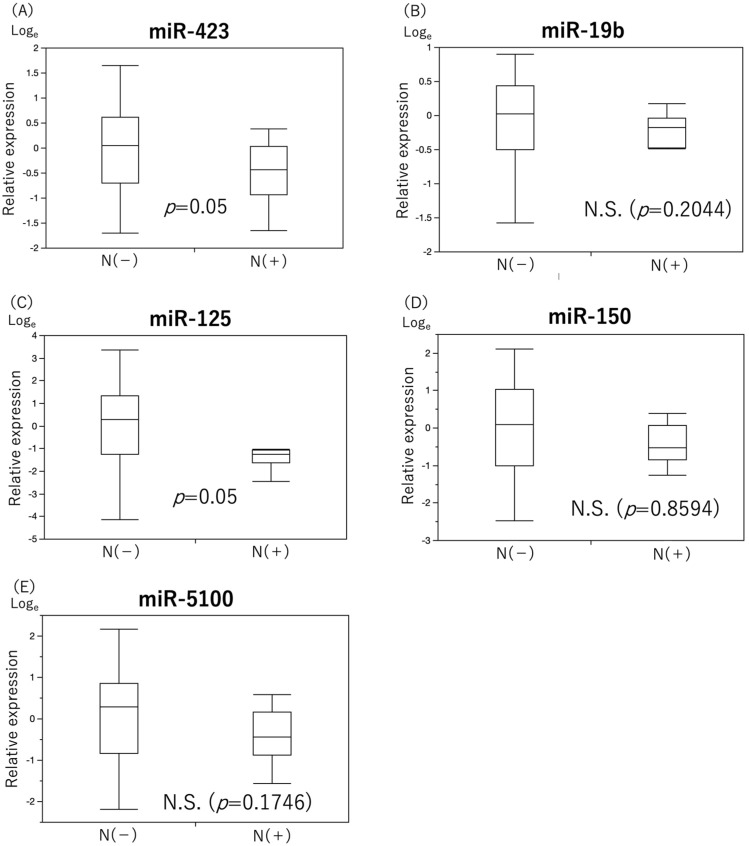
Comparison of expression levels of single miRNAs for primary neck lymph node metastases. The results of a comparison of the expression levels of five miRNAs that were useful for the diagnostic performance of primary neck lymph node metastasis in the groups with and without primary neck lymph node metastasis are shown ((**A**) miR-423, (**B**) miR-19b, (**C**) miR-125, (**D**) miR-150, (**E**) miR-5100). MiR-423 and miR-125 tended to be significantly down-regulated in the group with primary neck lymph node metastasis.

miR-19b, miR-150, and miR-5100 levels showed no significant differences, whereas the expression levels of miR-423 and miR-125 tended to be lower in the primary neck LN group (*p* = 0.05, Figure 2).

### 3.3. Diagnosis of Primary Neck LN Metastases Using Combination of Multiple miRNAs

Although the diagnostic performance of individual miRNAs for primary neck LN metastasis was relatively good, a regression equation for the diagnosis of primary neck LN metastasis was developed using linear analysis to calculate the miRNA index to diagnose primary neck LN metastasis with higher accuracy.

$$\begin{array}{c} \mathrm{miRNA}\;\mathrm{index}\;\mathrm{for}\;\mathrm{primary}\;\mathrm{metastasis}\;\mathrm{diagnosis}\;(\mathrm{miR-PM})\\ =2.15170+(-0.07994\times \mathrm{miR-23})+(-0.36912\times \mathrm{miR-19a})+(0.14258\times \mathrm{miR-4419a})\\ +(0.24627\times \mathrm{miR-5100}) \end{array}$$

**Figure 3:**
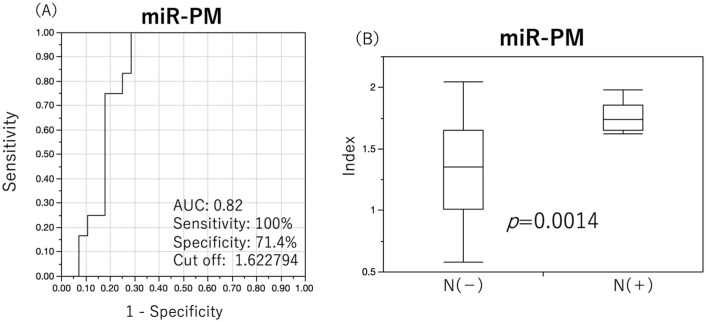
ROC analysis of miRNA index (miR-PM) and index differences. Diagnostic performance of miR-PM was investigated using ROC analysis to determine the cut-off value with the detection of primary neck lymph node metastasis as the outcome. MiR-PM showed a good diagnostic performance with an AUC of 0.82 (**A**). Comparison of the index of miR-PM between groups with and without primary neck lymph node metastases showed a significant increase in the group with primary neck lymph node metastases (**B**).

ROC analysis was performed using miR-PM to determine cut-off values. The diagnostic performance of miR-PM was examined based on the cut-off values from the analysis (Figure 3A), resulting in an AUC of 0.82, sensitivity of 100%, specificity of 71.4%, positive predictive value of 60.0%, negative predictive value of 100%, and Fisher’s exact test *p* value of 0.0001, showing a higher predictive performance than a single miRNA (Table 3). When comparing the difference in index values between the two groups, it was significantly increased in the primary neck LN metastasis group (*p* = 0.0014) (Figure 3B).

The diagnostic performance of serum SCC antigen and pathological factors for primary neck LN metastases in this study were statistically analyzed and compared with that of miR-PM (Table 3). YK classification had a sensitivity of 75.0%, specificity of 85.7%, positive predictive value of 60.0%, negative predictive value of 80.0%, and Fisher’s exact test *p* value of 0.04, which was relatively useful; however, the diagnostic performance of miRNAs was superior.

### 3.4. Prediction of Late Neck LN Metastases Using Single miRNA

**Figure 4:**
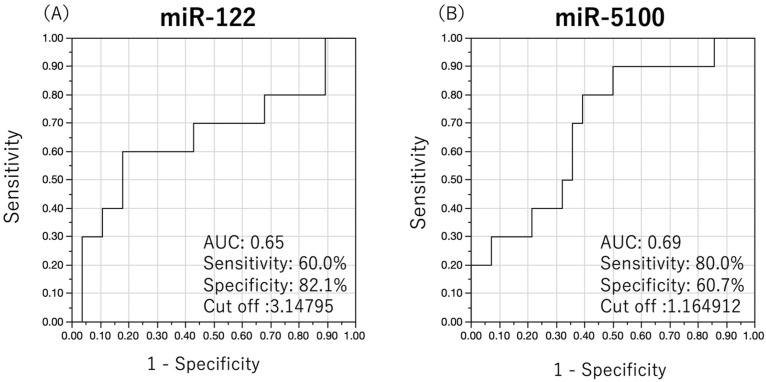
ROC analysis of late neck lymph node metastases using a single miRNA. Predictive performance of each miRNA was investigated by determining the cut-off value using ROC analysis, with the prediction of late neck lymph node metastasis as the outcome. ROC curves of the two miRNAs with useful predictive performance are shown. MiR-122 (**A**) and miR-5100 (**B**) showed good predictive performance with an AUC > 0.6.

We investigated whether quantitative measurement of OSCC-specific miRNAs in the serum of patients with OSCC without clinical LN metastasis at initial diagnosis could predict late neck LN metastasis. The predictive performance of each miRNA was examined using the cut-off values from the ROC analysis, with the prediction of late neck LN metastasis as the outcome. The ROC curves of the two miRNAs with useful predictive performance are shown in Figure 4, and the results of each analysis are shown in Table 4. miR-122 and miR-5100 showed good predictive performance with AUC values higher than 0.6. Results for the other 12 miRNAs are shown in the Supplementary Materials (Figure S3).

**Figure 5:**
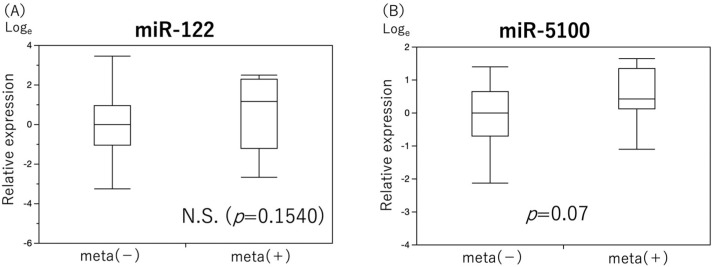
Comparison of expression levels of single miRNAs for prediction of late neck lymph node metastases. The results of a comparison of the expression levels of two miRNAs that were useful as predictors of late neck lymph node metastasis in groups with and without late neck lymph node metastasis are shown ((**A**) miR-122, (**B**) miR-5100). MiR-5100 tended to be significantly upregulated in the group with late neck lymph node metastasis.

miR-122 level showed no significant differences, whereas the expression level of miR-5100 tended to increase in the group with late neck LN metastases (*p* = 0.07, Figure 5).

### 3.5. Prediction of Late Neck LN Metastases Using Combination of Multiple miRNAs

Although the performance of a single miRNA in predicting late neck LN metastasis was relatively good, the following regression equation was established using linear analysis to predict late neck LN metastasis with increased accuracy.

$$\begin{array}{c} \mathrm{miRNA}\;\mathrm{index}\;\mathrm{for}\;\mathrm{late}\;\mathrm{metastasis}\;\mathrm{prediction}\;(\mathrm{miR-LM})\\ =-0.1753+(-0.5443\times \mathrm{miR-144})+(0.7120\times \mathrm{miR-5100}) \end{array}$$

**Figure 6:**
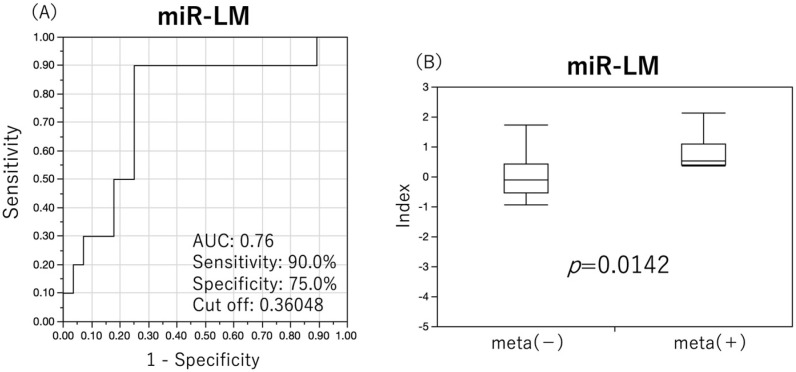
ROC analysis of miRNA index (miR-LM) and index differences. Predictive performance of miR-LM was investigated using ROC analysis to determine the cut-off value for the prediction of late neck lymph node metastasis as the outcome. MiR-LM showed a good predictive performance with an AUC of 0.76 (**A**). Comparison of the index of miR-LM between groups with and without late neck lymph node metastases showed a significant increase in the group with late neck lymph node metastases (**B**).

ROC analysis was performed using miR-LM to determine cut-off values. Based on the cut-off values from the analysis, the predictive performance of miR-LM was examined (Figure 6A). miR-LM showed an AUC of 0.76, sensitivity of 90.0%, specificity of 75.0%, positive predictive value of 56.25%, negative predictive value of 95.45%, and Fisher’s exact test *p* value of 0.0005, which was higher than the predictive performance values of individual miRNAs (Table 5). When comparing the difference in miR-LM between the two groups, it was significantly increased in the neck LN late metastasis group (*p* = 0.0142) (Figure 6B).

Comparisons were then made with the ability of serum SCC antigens and pathological factors to predict late neck LN metastasis (Table 5). Venous invasion showed a sensitivity of 60.0%, specificity of 75.0%, positive predictive value of 46.2%, negative predictive value of 84.0%, and Fisher’s exact test *p* value of 0.06, indicating a relatively useful predictive performance, although the predictive performance of miR-LM was superior.

### 3.6. Examination of miRNA, Serum SCC Antigen, Pathological Factors, and Survival Prognosis

**Figure 7:**
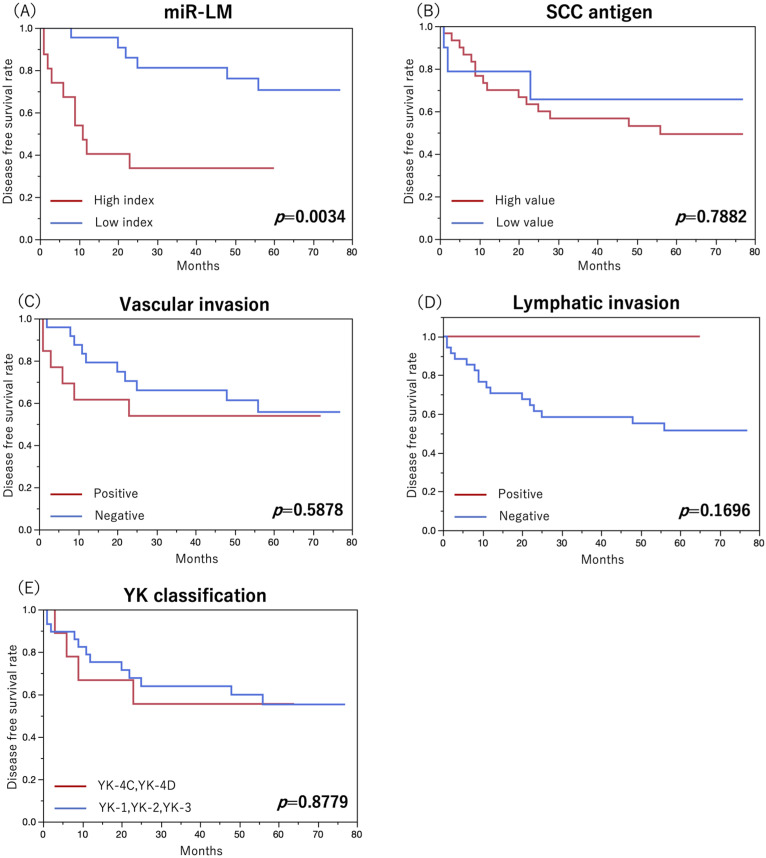
miRNAs, serum SCC antigens, pathological factors, and survival prognosis. To further investigate the prognostic performance, disease-free survival was examined using miR-LM (**A**), serum SCC antigen (**B**), and pathological factors (**C**–**E**). Results of Kaplan–Meier curves and log-rank tests are shown. Serum SCC antigen and pathological factors did not show statistically significant differences. miR-LM showed a statistically significant difference (*p* = 0.0034), suggesting that it is useful in predicting prognosis after treatment (five-year disease-free survival; low index group: 70.6%, high index group: 33.7%).

To further investigate the prognostic performance, DFS was examined using miR-LM, serum SCC antigen, and pathological factors. Results of Kaplan–Meier curves and log-rank tests are shown in Figure 7. No statistically significant differences were found for serum SCC antigen or pathological factors. miR-LM showed a statistically significant difference (*p* = 0.0034), suggesting that it is useful in predicting prognosis after treatment (five-year DFS; low index group: 70.6%, high index group: 33.7%).

## 4. Discussion

Metastasis is one of the most important factors influencing patient prognosis. The most common site of OSCC metastasis is the neck (neck LNs), and appropriate treatment of the neck LNs is essential for disease control. Appropriate biomarkers for OSCC have not been defined and diagnosis of neck LN metastases is dependent on imaging. Imaging studies are not performed frequently due to radiation exposure and the patient’s ability to metabolize contrast media, which can delay the detection of metastases, make treatment difficult, and often have life-threatening consequences depending on the timing. The diagnosis and prediction of neck LN metastasis, which is the most important factor in a patient’s prognosis, can be improved if LN metastasis can be treated early by detecting metastasis early using less-invasive biomarkers.

Biomarkers for the diagnosis and prognosis of disease must be easy to detect and minimally invasive to collect, the samples must be stable after collection, and their diagnostic and predictive performance must be highly accurate. Serum miRNAs can be collected from venous blood. miRNAs present in the blood are bound to vesicles and proteins and are not easily degraded [4]. Therefore, miRNAs specifically reflect the state of the lesion or damaged tissue. miRNAs are few in number compared to genes, with approximately 2500 different types. They are also easy to target therapeutically because they are directly involved in protein synthesis via mRNA degradation [3,22]. For these reasons, we have used miRNAs as biomarkers for OSCC.

For primary neck LN metastases, the diagnostic performance of miR-423 and miR-125 alone were useful, with a trend towards decreased expression in the primary neck LN metastasis group. For late neck LN metastases, the predictive performance of miR-5100 alone was useful, with a trend towards increased expression in the late neck LN metastasis group. In a previous report, we compared the expression levels of miRNAs in patients with OSCC and healthy subjects and found no change in the expression levels of these three miRNAs in the diagnostic performance of OSCC [17]. This suggests that miR-423 and miR-125 are specifically involved in neck LN metastasis and miR-5100 in late neck LN metastasis.

miR-423 is down-regulated in oral cancer tissue compared to normal tissue and acts as a tumor suppressor [23]. It has also been reported that the expression level of miR-423 in oral cancer is negatively correlated with T classification, neck LN metastasis, and stage classification, with a significantly decreased level in the neck LN metastasis group [24]. In osteosarcoma, miR-423 directly targets mRNA transcribed from the STMN1 (stathmin1) gene, which encodes stathmin, a protein responsible for the cytoskeleton. miR-423 overexpression was reported to suppress the growth and invasion of osteosarcoma cells [24]. In gastric cancer, miR-423 has been reported to enhance cell proliferation and invasive potential by targeting the tumor suppressor gene TFF1 (trefoil factor family 1) [25]. The present results suggest that tumor cells in groups with primary neck LN metastasis have reduced expression of miR-423, which may be involved in acquiring the functions and environment required for neck LN metastasis. However, there are insufficient reports of an association between miR-423 and LN metastasis in oral cancer, and no functional analysis has been performed. Further investigation is needed in the future.

In non-small cell lung cancer, miR-125 suppresses MMP expression by directly targeting mRNA transcribed from the MMP (matrix metalloproteinase) gene. MMPs play a role in the degradation of the extracellular matrix during cell division, differentiation, cell migration, angiogenesis, and apoptosis, and their aberrant expression in malignant tumors is responsible for tissue destruction. For these reasons, a negative correlation between miR-125 expression and LN metastasis and pathological stage has been reported [26]. In oral and head and neck cancers, miR-125 expression and carcinogenesis have been reported to be negatively correlated [27,28]. However, no association with neck LN metastasis has been reported; thus, further investigation, including functional analysis, is needed.

In oral cancer, miR-5100 has been reported to directly target the suppressor of cancer cell invasion (SCAI), which regulates cell migration and proliferative capacity and promote OSCC cell growth and invasion by suppressing SCAI [29]. It has been reported that serum miR-5100 expression is significantly increased in patients with metastatic lung cancer and may be a biomarker for predicting metastasis. This is because miR-5100 activates the STAT3 (Signal Transducers and Activators of Transcription 3) pathway and induces the expression of mesenchymal-associated molecules such as snail and vimentin. This has been reported to promote EMT in lung cancer cells, thereby promoting cell invasion [30]. In oral cancer, no direct association between miR-5100 and neck LNs has been reported. The effect of miR-5100 on OSCC is also unclear and requires further investigation.

Our previously reported study further improved the diagnostic performance of OSCC by examining a combination of multiple miRNAs rather than examining a single miRNA [17]. The present report similarly showed higher diagnostic and predictive performance by examining multiple miRNAs. Several combinations of miRNAs have been reported for the diagnosis and prognosis of oral cancer, with most having higher predictive performance than single miRNAs [31,32]. This may be because tumor tissue is composed of heterogeneous tumor cells, and the pathways involved in tumor cell growth, invasion, migration, and other functions are complex and involve many factors [33]. Therefore, multiple miRNAs are involved in tumor metastasis, and therefore, the prediction is accurate when multiple miRNAs are detected.

Serum SCC antigen is a biomarker for oral cancer in current use clinically; however, there is no definitive consensus on serum SCC antigen use and its clinical significance. It has been reported that serum SCC antigen is effective in predicting recurrent and metastatic cases, as its level is elevated prior to treatment [34]. In contrast, it has also been reported that SCC antigen is not correlated with recurrence or metastasis and is not a useful predictive marker for overall survival (OS) or DFS [35]. In the present results, there was no significant association between serum SCC antigen and primary or late neck LN metastasis.

The information obtained from pathology specimens directly reflects tumor characteristics. In particular, correlations between venous invasion, lymphatic invasion, type of invasion, and neck LN metastasis have been widely reported [10,11,36]. Such reports can be explained by the biological characteristics of the tumor tissue. Neoplasia of blood and lymphatic vessels is one of the characteristics of tumor tissue. Tumor cells invade blood or lymph vessels and travel through the bloodstream or lymphatic system to distant tissues to form metastases. This process is essential for tumor growth, invasion, and metastasis [37]. However, lesions are difficult to sample repeatedly, and serum biomarkers are needed to assess and predict a patient’s condition in real-time. The results of the present study showed that late neck LN metastasis could not be predicted using histopathology. However, serum miRNAs showed useful predictive performance for late neck LN metastasis.

There is no consensus on whether prophylactic neck dissection should be performed for potential neck LN metastases in oral cancer and what the predictive factors are. Many reports recommend prophylactic neck dissection for tongue cancer because of the high rate of potential neck LN metastases (26.8–48.2%) in cases of T2 or higher [10]. There are also reports that contralateral neck dissection should be performed in T3 or T4 cases if the primary tumor is close to the midline, even in cases with metastases in the affected neck LNs but no metastatic LNs on the contralateral side [38]. Pathological factors such as DOI, mode of invasion, and vascular invasion have been considered as predictors of potential neck LN metastases [24,39,40,41]. In particular, the NCCN guidelines for head and neck cancer (2022) recommend a DOI of 4 mm or more as an indication for prophylactic neck dissection. Our results show that miRNAs may be a predictor of potential neck LN metastasis in all sites of oral cancer and not just tongue cancer. In addition, miRNAs may be one of the factors to consider for prophylactic neck dissection.

One of the limitations of the present study was the small cohort size of patients with OSCC. The power of 0.99 for primary neck LN metastasis in the post hoc analysis from the G-Power results was considered statistically sufficient for the cohort size, whereas the power of 0.75 for late neck LN metastasis was considered slightly underpowering. In the future, prospective validation should be performed in another cohort group. In addition, we routinely collect not only preoperative blood samples, but also intraoperative and postoperative samples to examine differences in miRNA expression over time. By comparing the expression levels of miRNAs before and after surgery, we will be able to identify more accurate miRNAs as biomarkers. In addition, there are few reports analyzing miRNA functions in OSCC. Identification of prognostic miRNAs in patients with OSCC in clinical trials and functional analysis at the in vivo and in vitro level are essential for understanding the biological dynamics of oral cancer. The results could play an important role in identifying targets for drug therapy and improving the prognosis of patients with oral cancer.

We believe our report is novel and significant in the following respects. Inadequate follow-up is often cited as a limitation in other studies; however, in the present report, patients were followed-up for a long time, up to approximately 8 years. Preoperative serum samples were used to predict metastasis with limited preoperative information. The use of combination of several miRNAs for diagnosis reflects the biology of the tumor as a heterogeneous cell population. The results were more significant than those of serum SCC antigens, which are currently used as biomarkers clinically. Based on these results, miRNAs may be useful for diagnosing primary neck LN metastasis and predicting late neck LN metastasis and may help to consider personalized treatment, including surgical methods and postoperative treatment.

## 5. Conclusions

MiRNAs are useful for diagnosing primary neck LN metastases in OSCC and predicting late neck LN metastases. It may help to consider individualized treatment, including follow-up, surgical methods, and postoperative management.

## Figures and Tables

**Table 1:** Clinicopathological characteristics of the subject patients.

Variable	Patients (n = 40)
Age, mean (range)	67.3 (33–88)
Sex, n (%)	
Male	22 (55)
Female	18 (45)
Location, n (%)	
Tongue	21 (52.5)
Gingiva	14 (35)
Oral floor	4 (10)
Buccal mucosa	1 (2.5)
T classification, n (%)	
T1	4 (10)
T2	16 (40)
T3	7 (17.5)
T4	13 (32.5)
N classification, n (%)	
N0	28 (70)
N1	5 (12.5)
N2	7 (17.5)
N3	0 (0)
M classification, n (%)	
M0	40 (100)
M1	0 (0)
Stage classification, n (%)	
I	3 (7.5)
II	14 (35)
III	8 (20)
IV	15 (37.5)
Vascular invasion, n (%)	
(+)	14 (35)
(−)	26 (65)
Lymphatic invasion, n (%)	
(+)	3 (7.5)
(−)	37 (92.5)
Perineural invasion, n (%)	
(+)	7 (17.5)
(−)	33 (82.5)
YK classification, n (%)	
1	0 (0)
2	1 (2.5)
3	29 (72.5)
4C	6 (15)
4D	4 (10)
Local recurrence, n (%)	
(+)	7 (17.5)
(−)	33 (82.5)
Late metastasis in neck lymph nodes, n (%)	
(+)	10 (25)
(−)	28 (70)
Intra-area recurrence in neck lymph nodes	2 (5)

**Table 2:** Diagnostic performance of each miRNA for primary neck lymph node metastasis.

miRNA	Group	AUC	Sensitivity	Specificity	PPV	NPV	Positive	Negative	Fisher’s Test
			(%)	(%)	(%)	(%)	(n)	(n)	(*p*-Value)
miR-23	N(+)	0.63	58.3	71.4	46.7	80	7	5	0.0909
	N(−)						8	20	
miR-24	N(+)	0.54	33.3	89.3	57.1	75.8	4	8	0.168
	N(−)						3	25	
miR-423	N(+)	0.69	75	71.4	52.9	87	9	3	0.013
	N(−)						8	20	
miR-19a	N(+)	0.39	75	39.3	34.6	78.6	9	3	0.4844
	N(−)						17	11	
miR-19b	N(+)	0.63	83.3	57.1	45.5	88.9	10	2	0.0354
	N(−)						12	16	
miR-20a	N(+)	0.53	91.7	35.7	37.9	90.9	11	16	0.1244
	N(−)						18	16	
miR-22	N(+)	0.58	83.3	53.6	43.5	88.2	10	16	0.0408
	N(−)						13	16	
miR-122	N(+)	0.59	83.3	46.4	40	86.7	10	16	0.1523
	N(−)						15	16	
miR-125	N(+)	0.7	83.3	75	58.8	91.3	10	16	0.0012
	N(−)						7	16	
miR-144	N(+)	0.59	41.7	82.1	50	76.7	5	16	0.13294
	N(−)						5	16	
miR183	N(+)	0.63	66.7	64.3	44.4	81.8	8	16	0.0927
	N(−)						10	16	
miR-150	N(+)	0.6	75	57.1	42.9	84.2	9	16	0.008
	N(−)						12	16	
miR-4419a	N(+)	0.52	91.7	28.6	35.5	88.9	11	16	0.2332
	N(−)						20	16	
miR-5100	N(+)	0.64	83.3	53.6	43.5	88.2	10	16	0.0408
	N(−)						13	16	

Footnote: AUC: area under the curve; PPV: positive predictive value; NPV: negative predictive value; Fisher’s test: Fisher’s exact test.

**Table 3:** Diagnostic performance of serum SCC antigens, pathological factors, and miR-PM for primary neck lymph node metastases.

	Group	Sensitivity	Specificity	PPV	NPV	Positive	Negative	Fisher’s Test
		(%)	(%)	(%)	(%)	(n)	(n)	(*p*-Value)
serum SCC antigen	N(+)	16.7	71.4	20	66.7	2	10	0.6927
(cut-off 1.5)	N(−)					8	20	
Vascular invasion	N(+)	50	71.4	42.9	76.9	6	6	0.2808
	N(−)					8	20	
Lymphatic invasion	N(+)	8.3	92.9	33.3	70.3	1	11	1
	N(−)					2	26	
YK classification	N(+)	50	85.7	60	80	6	6	0.04
(YK1.2.3:positive,4c.4d:negative)	N(−)					4	24	
miR-PM	N(+)	100	71.4	60	100	12	0	0.0001
	N(−)					8	20	

Footnote: AUC: area under the curve; PPV: positive predictive value; NPV: negative predictive value; Fisher’s test: Fisher’s exact test.

**Table 4:** Predictive performance of each miRNA for late neck lymph node metastasis.

miRNA	Group	AUC	Sensitivity	Specificity	PPV	NPV	Positive	Negative	Fisher’s Test
			(%)	(%)	(%)	(%)	(n)	(n)	(*p*-Value)
miR-23	meta(+)	0.56	100	21.4	31.3	100	10	0	0.1679
	meta(−)						22	6	
miR-24	meta(+)	0.58	100	35.7	35.7	100	10	0	0.0377
	meta(−)						18	10	
miR-423	meta(+)	0.52	100	25	32.2	100	10	0	0.1564
	meta(−)						21	7	
miR-19a	meta(+)	0.47	100	10.7	28.6	100	10	0	0.5519
	meta(−)						25	3	
miR-19b	meta(+)	0.46	100	17.9	30.3	100	10	0	0.2984
	meta(−)						23	5	
miR-20a	meta(+)	0.56	100	32.1	34.5	100	10	0	0.0785
	meta(−)						19	9	
miR-22	meta(+)	0.39	90	25	30	87.5	9	1	0.6533
	meta(−)						21	7	
miR-122	meta(+)	0.65	60	82.1	54.5	85.2	6	4	0.0193
	meta(−)						5	23	
miR-125	meta(+)	0.5	90	32.1	32.1	90	9	1	0.2362
	meta(−)						19	9	
miR-144	meta(+)	0.52	40	82.1	44.4	79.3	4	6	0.2051
	meta(−)						5	23	
miR183	meta(+)	0.51	100	14.3	29.4	100	10	0	0.5562
	meta(−)						24	4	
miR-150	meta(+)	0.49	40	85.7	50	80	4	6	0.1701
	meta(−)						4	24	
miR-4419a	meta(+)	0.54	40	85.7	50	80	4	6	0.1701
	meta(−)						4	24	
miR-5100	meta(+)	0.69	80	60.7	45	85	8	2	0.0625
	meta(−)						11	17	

Footnote: AUC: area under the curve; PPV: positive predictive value; NPV: negative predictive value; Fisher’s test: Fisher’s exact test.

**Table 5:** Predictive performance of serum SCC antigens, pathological factors, and miR-LM for late neck lymph node metastases.

	Group	Sensitivity	Specificity	PPV	NPV	Positive	Negative	Fisher’s Test
		(%)	(%)	(%)	(%)	(n)	(n)	(*p*-Value)
serum SCC antigen	meta(+)	30	75	30	75.7	3	7	1
(cut-off 1.5)	meta(−)					7	21	
Vascular invasion	meta(+)	60	75	46.2	84	6	4	0.06
	meta(−)					7	21	
Lymphatic invasion	meta(+)	0	89.9	0	71.4	0	10	0.5519
	meta(−)					3	25	
YK classification	meta(+)	40	82.1	44.4	79.3	4	6	0.2051
(YK1.2.3:positive YK4c.4d:negative)	meta(−)					5	23	
miRNA index (miR-LM)	meta(+)	90	75	56.3	95.5	9	1	0.0005
	meta(−)					7	21	

Footnote: AUC: area under the curve; PPV: positive predictive value; NPV: negative predictive value; Fisher’s test: Fisher’s exact test.

## Data Availability

Data available on request due to restrictions of ethical policy.

## Supplementary Materials

The following supporting information can be downloaded at: https://www.mdpi.com/article/10.3390/cancers15153769/s1, Figure S1. The typical histology that was determined to be lymphatic and vascular invasion and the representative histology for each of the YK classifications in the present study. Figure S2: ROC analysis for primary neck lymph node metastases using a single miRNA; Figure S3. ROC analysis for late neck lymph node metastases by a single miRNA. Table S1: List of miRNAs used for RT-PCR.
