# Supplementary material for: Identification of Neck Lymph Node Metastasis-Specific microRNA—Implication for Use in Monitoring or Prediction of Neck Lymph Node Metastasis
Source: Cancers (Basel). 2023 Jul 25;15(15):3769. doi: 10.3390/cancers15153769 (PMC10417354; doi:10.3390/cancers15153769)
Supplement: Supplementary file 1 [file cancers-15-03769-s001.zip › cancers-2495616-supplementary.pdf]

cancers

MDPI

Supplementary Materials

Identification of Neck Lymph Node Metastasis-Specific microRNA—Implication for Use in Monitoring or Prediction of Neck Lymph Node Metastasis

Yutaro Higashi [1,2], Kodai Nakamura [1], Ryota Takaoka [1], Mika Tani [1], Yusaku Noma [1], Kazuki Mori [1], Kota Yamashiro [1], Seiya Yokoyama [3], Tomofumi Hamada [4,*] and Tsuyoshi Sugiura [1,2,*]

1 Department of Maxillofacial Diagnostic and Surgical Science, Field of Oral and Maxillofacial Rehabilitation, Graduate School of Medical and Dental Science, Kagoshima University, Kagoshima 890-8544, Japan; yutaro.higashi.b6@tohoku.ac.jp (Y.H.); pero-pulse-4.14@true.ocn.ne.jp (K.N.); k7506163@kadai.jp (R.T.); k1946287@kadai.jp (M.T.); k7794925@kadai.jp (Y.N.); k5538059@kadai.jp (K.M.); yama19@dent.kagoshima-u.ac.jp (K.Y.)

2 Division of Oral and Maxillofacial Oncology and Surgical Sciences, Tohoku University Graduate School of Dentistry, Sendai 980-8575, Japan

3 Department of Pathology, Kagoshima University Graduate School of Medical and Dental Sciences, Kagoshima University, Kagoshima 890-8544, Japan; yokoyama@m3.kufm.kagoshima-u.ac.jp

4 Department of Oral & Maxillofacial Surgery, Hakuaikai Medical Cooperation Sagara Hospital, Kagoshima 892-0833, Japan

* Correspondence: thamada@sagara.or.jp (T.H.); tsuyoshi.sugiura.b2@tohoku.ac.jp (T.S.); Tel.: +81-99-224-1800 (T.H.); +81-22-717-8350 (T.S.)

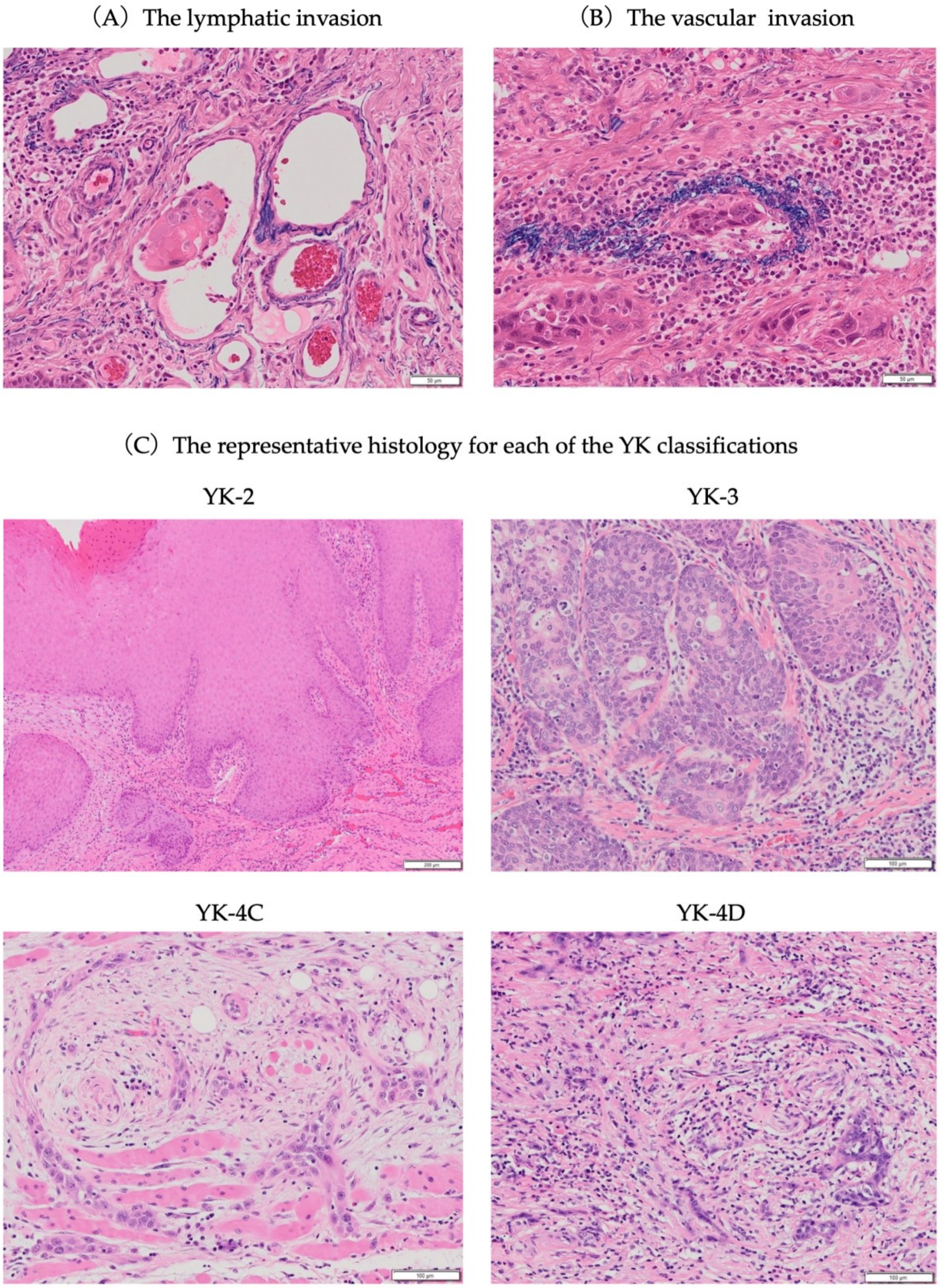

Figure S1. The typical histology that was determined to be lymphatic and vascular invasion and the representative histology for each of the YK classifications in the present study. The typical histology of patients with OSCC in the study is shown. (A) and (B) were stained with Victoria blue-hematoxylin-eosin. (C) was stained with hematoxylin-eosin.

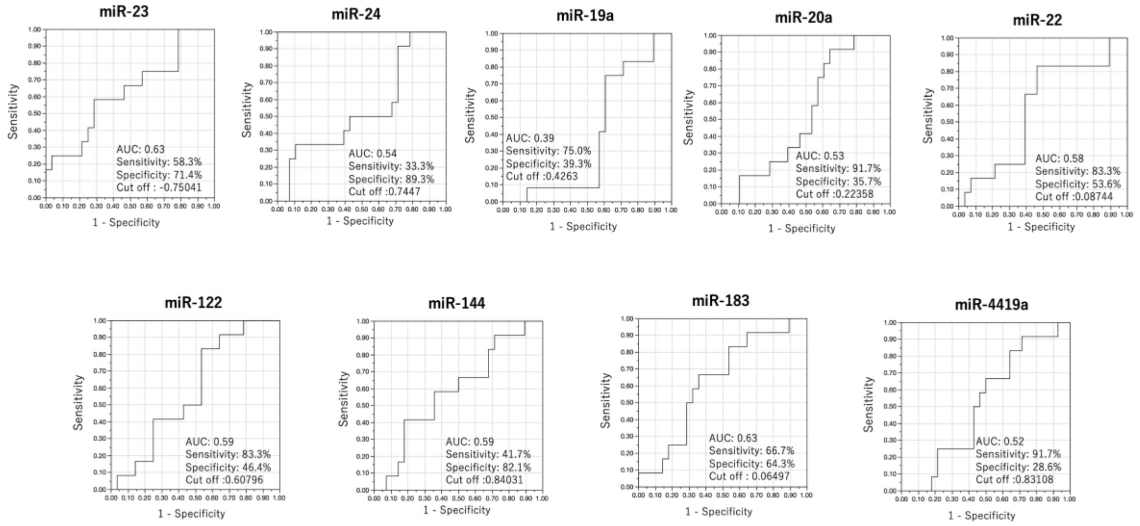

Figure S2. ROC analysis for primary neck lymph node metastases by a single miRNA. Diagnostic performance of each miRNA was investigated by determining the cut-off value by ROC analysis, with the detection of primary neck lymph node metastasis as the outcome.

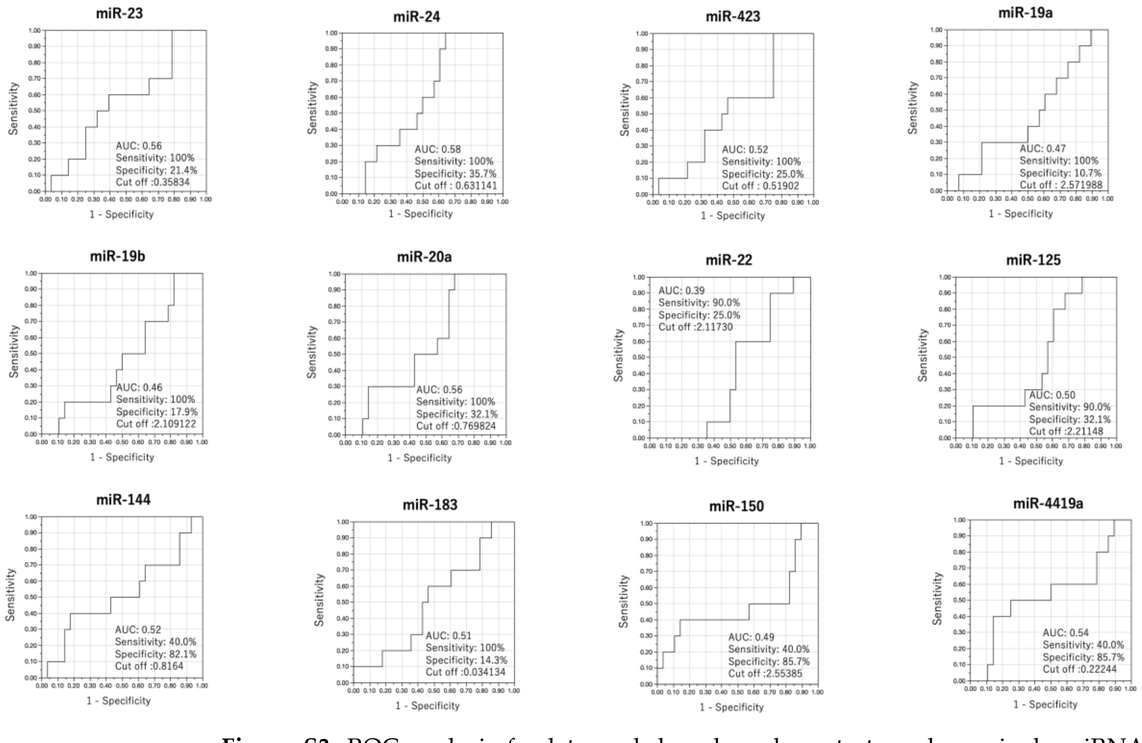

Figure S3. ROC analysis for late neck lymph node metastases by a single miRNA. **P**redictive performance of each miRNA was investigated by determining the cut-off value by ROC analysis, with the prediction of late neck lymph node metastasis as the outcome.

Table S1. List of miRNAs used for RT -PCR.

miRNA
miR-23a-3p
miR-24-3p
miR-423-5p
miR-19a-3p
miR-19b-3p
miR-20a-5p
miR-22-3p

miR-122-5p
miR-125-2-3p
miR-144-3p
miR-183-5p
miR-150-3p
miR-4419a
miR-5100
